# One-Stage Hybrid Surgery for Complicated Acute Type B Aortic Dissection Involving Distal Arch Aneurysm: A Case Report

**DOI:** 10.3400/avd.cr.25-00039

**Published:** 2025-08-20

**Authors:** Kenji Kishita, Naoki Washiyama, Yuki Takeuchi, Masahiro Hirano, Ken Yamanaka, Yuko Ohashi, Kazumasa Tsuda, Kazuma Okamoto

**Affiliations:** Department of Surgery 1, Division of Cardiovascular Surgery, Hamamatsu University School of Medicine, Hamamatsu, Shizuoka, Japan

**Keywords:** complicated acute type B aortic dissection, distal arch aortic aneurysm, thoracic endovascular aortic repair

## Abstract

An 84-year-old woman with an acute type B aortic dissection (ATBAD), an entry tear in a distal arch aneurysm, and lower-body malperfusion underwent a hybrid approach combining total arch replacement with an elephant trunk (TAR+ET), thoracic endovascular aortic repair (TEVAR), and left renal artery stenting. This strategy avoided direct resection of the aneurysm or primary entry, yet stabilized hemodynamics and restored organ perfusion. Postoperative CT was favorable, and the patient was discharged without complications. In this elderly case of complicated ATBAD involving a distal aortic arch aneurysm, we performed TAR+ET, TEVAR, and renal artery stenting, and achieved a favorable outcome.

## Introduction

Acute type B aortic dissection is often managed with stringent blood pressure control, but there are also cases in which emergency surgery is required.^1)^ When malperfusion necessitates emergent intervention, recent reports have shown that therapy should be tailored to the mechanism of obstruction: thoracic endovascular aortic repair (TEVAR) aimed at closing the entry tear for dynamic obstruction and branch stenting or peripheral bypass for static obstruction.^2)^ We encountered a patient with an acute type B aortic dissection complicated by lower-body malperfusion requiring urgent surgery. Because the entry tear was in the pre-existing distal aortic arch aneurysm, simply closing the primary entry using TEVAR could not be performed easily. Graft replacement using open proximal anastomosis through left thoracotomy is one option, but surgery via left thoracotomy is not feasible at every institution, and in emergency cases, it may have to be performed by surgeons who are not accustomed to that approach. Furthermore, we were concerned preoperatively that central repair alone would not improve the renal artery ischemia, reflecting the complex nature of this pathology. Therefore, we combined total arch replacement (TAR), TEVAR, and stent placement in the renal artery.

## Case Report

An 84-year-old female with a history of cerebral infarction, hypertension, and appendicitis presented with a sudden onset of chest and back pain. She had been independent in activities of daily living. After a walk, she felt unwell and lay down to rest, gradually developing left-sided back pain that intensified and prompted her visit to our emergency department.

On admission, blood pressure was 173/86 mmHg in the right arm, 173/65 mmHg in the left arm, 70/49 mmHg in the right leg (Ankle–Brachial Index (ABI) = 0.4), and 110/75 mmHg in the left leg (ABI = 0.65). She had no heart murmurs or abnormal lung sounds, a soft and flat abdomen, numbness in the left foot, and persistent pain in the left back. Bilateral dorsalis pedis pulses were not palpable. Echography suggested decreased flow through the superior mesenteric artery.

**Figure 1:**
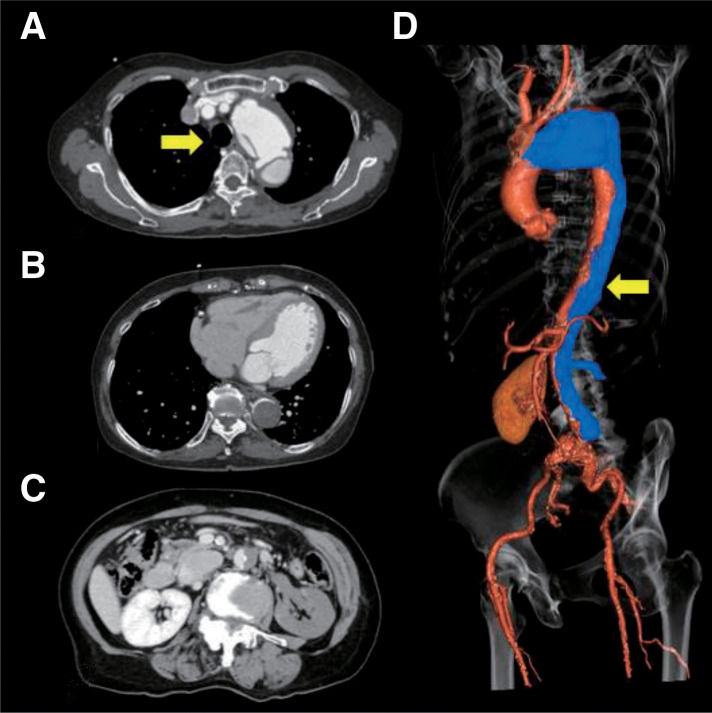
Fig. 1 Preoperative CT scan. Preoperative contrast-enhanced CT demonstrated an acute type B aortic dissection with a patent false lumen within the distal aortic aneurysm (**A**, arrow). The false lumen within the aneurysm remained patent. At the same time, the mid-descending thoracic aorta and distal segments exhibited a thrombosed false lumen type, and the true lumen below the descending aorta was compressed (**B**). The left renal artery was not enhanced in the late phase (**C**). The arrow indicates the false lumen in the 3D reconstructed image (**D**). 3D: 3 dimensional; CT: computed tomography

Blood tests revealed lactate dehydrogenase (LDH) 195 U/L, creatine kinase (CK) 75 U/L, creatinine 0.63 mg/dL, N-terminal pro-brain natriuretic peptide (NT-proBNP) 84 pg/mL, hemoglobin 12.6 g/dL, platelet count 19.2 × 10^4^/μL, prothrombin time/international normalized ratio (PT-INR) 1.06, activated partial thromboplastin time (APTT) 34.6 s, D-dimer 24.1 μg/mL, and lactate 1.20 mmol/L on venous blood gas analysis. Electrocardiography showed a heart rate of 51 bpm with sinus rhythm and no ST changes. A chest radiograph showed a prominence of the left aortic knob. Contrast-enhanced CT revealed aortic dissection extending from the origin of the left subclavian artery to above the ordinary iliac artery bifurcation level. A distal aortic aneurysm measuring 62 mm in maximum minor diameter was identified, containing an entry tear. The ascending aorta had a maximum diameter of 39.5 mm. The false lumen remained patent from the distal arch through the mid-descending thoracic aorta, while the true lumen of the lower-descending and the abdominal aorta was markedly narrowed. The celiac artery, superior mesenteric artery, and right renal artery originated from the true lumen, whereas the left renal artery and inferior mesenteric artery arose from the false lumen. Consequently, the left kidney showed poor contrast enhancement (**Fig. 1**).

**Figure 2:**
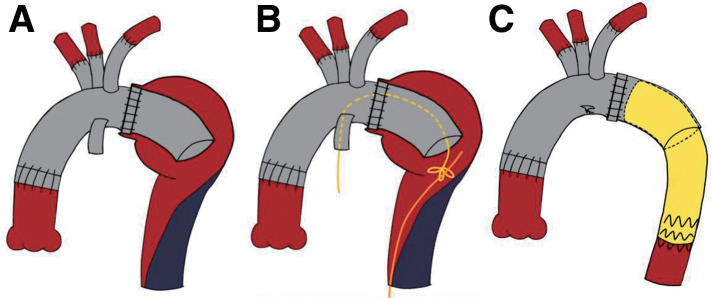
Fig. 2 Schematic illustration of TEVAR technique during surgery. Similarly, the length of the ET was set to 5 cm to remain within the distal arch aneurysm (**A**). The snare wire was then advanced through a side branch of a 4-branched graft into the distal side, and the guidewire introduced from the femoral artery was captured by the snare wire, completing the pull-through technique (**B**). Finally, TEVAR was performed using the ET as the landing zone, resulting in an expanded true lumen in the descending aorta (**C**). ET: elephant trunk; TEVAR: thoracic endovascular aortic repair

These findings indicated acute type B dissection accompanied by lower-body malperfusion that necessitated emergent surgery. Malperfusions other than the left renal artery were classified as a dynamic obstruction. Because of the distal arch aneurysm, conventional TEVAR was not anatomically feasible. As the entry tear was located within the distal arch aneurysm and the false lumen below the lower-descending aorta was thrombosed, we anticipated that sealing the distal side of the arch aneurysm would eliminate the false lumen flow within it. Thus, our central repair plan involved TAR via zone 2 distal anastomosis and placement of a conventional elephant trunk (ET) within the aneurysm, followed by TEVAR using the ET as the proximal landing zone after weaning from cardiopulmonary bypass (**Fig. 2**). If subsequent angiography still showed inadequate perfusion of the left renal artery, a stent would be placed in the renal artery.

The procedure was initiated in a hybrid operating room equipped with a C-arm. We established a cardiopulmonary bypass by the ascending aorta and the right femoral artery and initiated systemic cooling. At 25°C deep hypothermia, we arrested systemic circulation and began selective antegrade cerebral perfusion. A 4-branched vascular graft with a diameter of 24 mm (J graft; Japan Lifeline, Tokyo, Japan) was used for the distal anastomosis along with an ET with a diameter of 24 mm. The ET measured 5 cm along the greater curvature and 4 cm along the lesser curvature, ensuring its distal end remained within the aneurysm. We reinitiated systemic circulation through a graft side branch to rewarm. After completing the proximal anastomosis and removing the aortic clamp, we reconstructed the 3 arch vessels, achieved hemostasis, and discontinued cardiopulmonary bypass. We advanced a snare wire from the branch that had been used for perfusion and caught a wire inserted from the left common femoral artery to create a pull-through system. Through the left common femoral artery, we deployed a 26 mm × 20 cm C-TAG stent graft (W. L. Gore & Associates, Newark, DE, USA). After TEVAR, contrast imaging revealed that the aortic stenosis had been relieved. However, the left renal artery did not opacify, leading to a diagnosis of static obstruction. Given its lower profile and the lower risk of thrombotic occlusion compared with a stent graft, we opted to use a bare-metal stent and attempted deployment from the true lumen. S.M.A.R.T. stents (6 mm × 4 cm and 6 mm × 6 cm) (Cordis, Miami Lakes, FL, USA), which are self-expanding bare-metal stents, were inserted into the left renal artery, and blood flow was restored.

**Figure 3:**
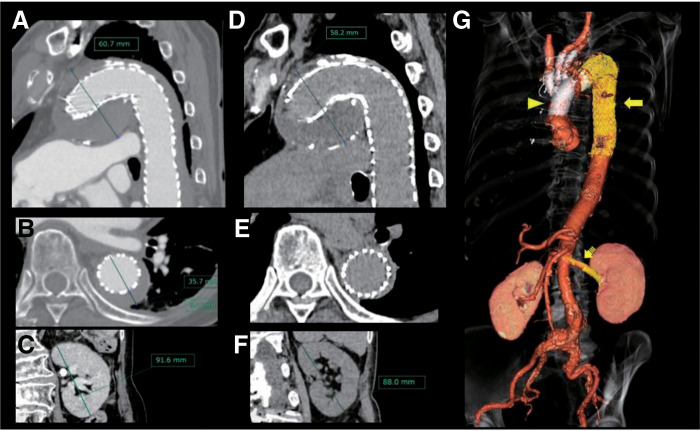
Fig. 3 CT image at 1 week and 3 months postoperatively; 3D reconstruction at 1 week postoperatively. On the postoperative CT image obtained on day 7, the maximum short-axis diameter of the distal arch aneurysm measured 60.7 mm (**A**), and the false lumen at the Th8 level measured 9.0 mm (**B**). At 3 months postoperatively, the maximum short-axis diameter of the distal arch aneurysm had slightly reduced to 58.2 mm (**D**), and the false lumen at the Th8 level had almost disappeared (**E**). The 3D reconstructed image of the CT on postoperative day 7 shows the triangle representing the prosthetic graft used for TAR+ET, and the arrow indicates the TEVAR stent. The dotted arrow shows the stent in the left renal artery, demonstrating contrast enhancement in the left kidney (**G**). In the delayed phase of the contrast-enhanced CT performed on postoperative day 7, the left kidney demonstrated contrast enhancement. (**C**), and no significant changes in kidney size or morphology were observed at 3 months (**F**). 3D: 3 dimensional; CT: computed tomography; ET: elephant trunk; TAR: total arch replacement; TEVAR: thoracic endovascular aortic repair

The operation lasted 523 min, including 193 min of cardiopulmonary bypass, 92 min of cardiac arrest, 71 min of aortic cross-clamping, 112 min of selective cerebral perfusion, 45 min of systemic circulatory arrest, and 5 min of total circulatory arrest. Blood loss was 2483 mL, and transfusions comprised 12 units of packed red blood cells, 10 units of fresh frozen plasma, and 30 units of platelet concentrates (totaling 3480 mL). The total administered iodine dose was 69.8 mL. Postoperatively, the patient returned to the intensive care unit (ICU) under mechanical ventilation. She was extubated on the 1st postoperative day without evidence of cerebral infarction or spinal cord injury (SCI). On postoperative day 7, a contrast-enhanced CT scan showed no anastomotic leaks (**Fig. 3A**), an adequately expanded stent graft in the distal portion, no endoleak (**Fig. 3B**), and restored perfusion of the left renal artery (**Figs. 3C** and **3E**). The peak serum creatinine was 1.16 mg/dL, decreasing to 0.74 mg/dL at discharge. She was able to walk 100 m at discharge independently, but her oral intake remained inadequate. On postoperative day 20, she was transferred to a rehabilitation hospital. After rehabilitation, the patient has returned to living at home. At 3 months postoperatively, plain CT showed that the distal arch aneurysm had shrunk to 58.2 mm (**Fig. 3D**), and the false lumen at the Th8 level had almost disappeared (**Fig. 3E**). Furthermore, no significant changes in kidney size or morphology were observed on 3-month non-contrast CT (**Fig. 3F**).

## Discussion

We report a case of acute type B aortic dissection with a distal arch aneurysm and lower-body malperfusion. TEVAR and central surgical repair aimed at entry closure are well-known strategies for treating complicated acute type B aortic dissection.^3)^ Branched/fenestrated TEVAR has become widespread in recent years, and given the advanced age of many patients, it is now a viable treatment option.^4)^ Moreover, a left thoracotomy procedure allows simultaneous aneurysm resection and relief of aortic obstruction. However, neither branched/fenestrated TEVAR nor left thoracotomy is available at every center, even in emergency situations. TAR can be performed even in emergency situations when a surgeon experienced in left thoracotomy is not available. We explored 2 approaches: TAR with frozen ET (TAR-FET) or conventional ET. Although aortic occlusion could have been relieved by TAR-FET, definitively relieving the occlusion would have required extending the stent graft down to approximately the T9 vertebral level. Deploying a stent graft distally posed a high risk of SCI. TEVAR offers 2 major advantages over FET. First, in a study comparing FET with conventional ET repair followed by 2-staged TEVAR, the latter demonstrated a significantly lower incidence of SCI [TAR-FET 16.7% (n = 9/54), 2-staged conventional ET+TEVAR 0% (n = 0/37)],^5)^ indicating that this strategy offers an additional advantage in terms of spinal cord protection. Second, a previous report has shown that the incidence of distal stent-induced new entry tear (dSINE) was 12.7% (n = 69/544)^6)^ with FET and 6.2% (n = 53/852) with TEVAR, and multivariate analysis showed that, in TEVAR, a stent graft length <165 mm was associated with the occurrence of dSINE.^7)^ As is this surgical technique, its lower dSINE risk compared to FET is also an advantage. Therefore, we decided to perform TEVAR using the conventional ET as the landing zone; by placing the stent graft under restored circulation, we believed we could reduce the risk of SCI. Furthermore, with TEVAR we could directly confirm that the aortic occlusion was relieved and accurately determine the position of the distal end of the stent, so we chose TEVAR. Although there are previous reports of combining conventional ET with 2-stage TEVAR for true distal arch aneurysms,^8,9)^ no cases have been reported using this combined approach in a single stage for acute aortic dissection.

Conventionally, TAR with ET and distal anastomosis beyond the distal arch aneurysm in zone 4 have been employed for similar cases. However, performing distal anastomosis on fragile dissected tissue located deep in the chest posed significant technical challenges, including prolonged circulatory arrest, risk of unexpected bleeding, and concerns about inadequate actual lumen expansion due to anastomotic leakage. To reduce technical complexity, we considered positioning the distal anastomosis of the TAR in zone 2. However, in TAR-FET, even using the maximum available length (15 cm) for distal anastomosis at zone 2, the distal end of the FET would align with the sharply curved region of the distal arch aneurysm, raising concerns about stent kinking and SINE caused by spring-back force from zone 2 anastomosis. Additionally, since false lumen flow ceased at the T8 vertebral level, repair needed to extend precisely to this point, making conventional ET combined with TEVAR a more suitable option than FET.

We decided to place the ET within the distal arch aneurysm and deploy TEVAR immediately after the completion of TAR and restoration of a spontaneous heartbeat. During TEVAR, to avoid inadvertent guidewire entry into the false lumen from the femoral artery, we advanced a snare wire antegradely via a perfusion branch of the 4-branched graft used for arch replacement, securely guiding the femoral artery-introduced guidewire into the true lumen. Although deploying TEVAR during circulatory arrest is an alternative option, in this case, we completed distal and proximal arch anastomoses along with cervical branch reconstruction, restarted circulation, restored spontaneous heartbeat, discontinued cardiopulmonary bypass, and subsequently deployed TEVAR. This method aimed to minimize extracorporeal circulation and hypothermic circulatory arrest times, enabling more precise stent positioning and sizing, thus reducing the risk of distal SINE.

In this case, the most urgent issues were critical aortic narrowing. One could consider performing only TEVAR (depending on the facility, anti-anatomical bypass) with or without renal artery stenting initially, followed by elective, 2-stage resection of the distal arch aneurysm. However, there have been reports of rapid aneurysm expansion when acute dissection occurs in the setting of a pre-existing aortic aneurysm,^10)^ and it was felt that reducing false-luminal volume via TEVAR might increase false-lumen pressure within the distal arch aneurysm, precipitating such accelerated enlargement. For this reason, a 1-stage treatment approach was chosen. Even in cases of extreme urgency, such as aortic occlusion and elevated lactate levels, this strategy can maintain blood flow to the lower body by delivering femoral artery flow, and may be an option. However, depending on the size of the aneurysm, TEVAR alone followed by 2nd operation may be better in cases of extreme urgency.

Pre-existing dynamic obstruction from the aortic dissection flap improved following central repair with TAR-ET plus TEVAR. In contrast, left renal artery malperfusion, involving both static and dynamic components, did not improve with false lumen decompression alone, necessitating additional renal artery stenting in combination with TAR and TEVAR.

A limitation of our approach was neither excising the distal arch aneurysm nor closing the entry. However, preoperative CT revealed no branches from the distal arch aneurysm that might cause type 2 endoleaks, and the communication between true and false lumens was limited to 1 site distal to the left subclavian artery. Additionally, postoperative CT demonstrated substantial thrombosis in the distal false lumen with minimal blood inflow. Thus, barring type 1 endoleaks or distal SINE, sufficient aneurysm decompression could be achieved, anticipating a favorable long-term prognosis. Furthermore, because intraoperative angiography is required for this procedure, a hybrid operating room is suitable.

## Conclusion

In an elderly case of complicated acute type B aortic dissection involving a distal aortic arch aneurysm, we performed TAR plus an ET to create a proximal landing zone, followed by TEVAR, combined with renal artery stenting for relief of static obstruction, and achieved a favorable outcome.

## Declarations

### Informed consent

The patient provided informed consent for the study of her case details and images.

### Disclosure statement

The authors have no conflicts of interest to disclose.

### Author contributions

Study conception: KO

Data collection: KK

Analysis: KK

Manuscript preparation: KK

Funding acquisition: KO and NW

Critical review and revision: all authors

Final approval of the article: all authors

Accountability for all aspects of the work: all authors.
